# One year symptom severity and health-related quality of life changes among Black African patients undergoing uterine fibroid embolisation

**DOI:** 10.1186/s13104-017-2558-0

**Published:** 2017-07-04

**Authors:** Charles Mariara, Timona Obura, Nigel Hacking, William Stones

**Affiliations:** 1 Department of Obstetrics and Gynaecology, AIC Kijabe Hospital, Kijabe, Kenya; 2 Department of Obstetrics and Gynaecology, Aga Khan University, Nairobi, Kenya; 3 Department of Radiology, University Hospital Southampton NHS Foundation Trust, Southampton, SO16 6YD UK; 4 Department of Obstetrics & Gynaecology, Malawi College of Medicine, Blantyre, Malawi; 5 Departments of Public Health and Obstetrics & Gynaecology, Malawi College of Medicine, Blantyre, Malawi; 6 St George’s, University of London, Molecular & Clinical Sciences Research Institute, London, SW17 0RE UK

**Keywords:** Uterine fibroid embolisation (UFE), Uterine fibroid symptom and quality of life questionnaire (UFS-QOL), Health-related quality of life (HRQOL), Symptom severity

## Abstract

**Background:**

The main aim in the treatment of symptomatic fibroids by various modalities including uterine fibroid embolisation (UFE) is to alleviate symptoms and ultimately improve the quality of life. The efficacy of this modality of treatment in Black African women with significant fibroid burden and large uterine volumes is not clear. The main objective of the study was to examine potential changes in symptom severity among Black African patients 1 year following UFE for symptomatic uterine fibroids in a resource-constrained setting, rated using a validated questionnaire (UFS-QOL). Secondary outcomes examined were changes in quality of life and potential associations with age, parity, uterine volume and fibroid number prior to UFE. Additional interventions after UFE were also recorded.

**Methods:**

A prospective before and after study of Black African patients undergoing UFE was undertaken. Participants underwent pelvic MR imaging prior to UFE and completed the UFS-QOL, a validated condition-specific questionnaire at baseline and at 1 year. Ninety five participants were recruited and data from 80 completing 1 year of follow up were available for analysis of changes in the symptom severity scores.

**Results:**

The mean reduction in symptom severity score was 29.6 [95% CI 23.6 to 35.6, P < 0.001] and the mean improvement in HRQOL score was 35.7 [95% CI 28.4 to 42.9, P < 0.001]. A greater number of fibroids identified prior to UFE was associated with a more substantial improvement in symptom severity score (r_s_ = 0.28, n = 80, P = 0.013) and participants of higher parity reported a greater improvement in HRQOL score (r = 0.336, P = 0.002). Major and minor surgical interventions were needed in 5 (6.3%) and 10 (12.5%) participants respectively.

**Conclusions:**

UFE is associated with clinically useful and statistically significant symptom relief in Black African patients. Symptom improvement following UFE is not compromised by a large fibroid burden and the rate of subsequent intervention is within an acceptable range. UFE is a safe alternative and efforts are needed to widen access to this non-surgical treatment modality.

**Electronic supplementary material:**

The online version of this article (doi:10.1186/s13104-017-2558-0) contains supplementary material, which is available to authorized users.

## Background

Uterine leiomyomas (fibroids) are the commonest solid non-cancerous tumors in women of the reproductive age group [1] and more than half are asymptomatic [2]. Where symptoms prompt clinical presentation this is typically for heavy or prolonged menstrual bleeding, pelvic pain and pressure symptoms. Fibroids may also be a feature in sub-fertility and miscarriage but the relationship between fibroid disease and sub-fertility may be coincidental rather than causal [3]. Racial differences as regards incidence and severity of uterine fibroids has been documented with women of African descent being noted to be approximately three times more likely to have fibroids compared to their Caucasian counterparts [4]. Studies have demonstrated that women of African descent compared to their Caucasian counterparts present at a younger age and have more symptoms. Women of African descent have bigger and more numerous fibroids with a faster rate of growth [5]. Age related reduction in the rate of fibroid growth is seen in Caucasian women but not in those of African descent [6]. The pathophysiological basis for such clinical observations is unclear and given the lack of clear biological definitions of race or ethnicity they should not be over emphasized, especially as there is considerable variation in the clinical manifestations of fibroids between individuals as well as between groups.

Pharmacological interventions in the treatment of symptomatic uterine fibroids usually have short term effects, some with significant associated costs and adverse effects [7]. The principal surgical modalities include hysterectomy and myomectomy. Hysterectomy constitutes a ‘cure’ but is naturally unacceptable to women who desire future childbearing. Myomectomy on the other hand is a major surgical procedure associated with morbidity and appreciable mortality risk even in well-resourced health care settings [8]. These hazards are especially problematic in resource-constrained setting where there may be limited access to blood transfusion. Uterine fibroid embolisation (UFE) is a radiological intervention that has increasingly been used in the treatment of symptomatic uterine fibroids by interruption of blood flow in the uterine arteries which occludes blood supply to the fibroid but not to normal myometrium owing to the presence of many collaterals [9]. While fibroid embolisation has become established as a standard treatment option in Western countries, its efficacy and safety together with understanding of cost considerations in the African setting, which has a substantial burden of fibroid related disease is not clear. We aimed to examine potential changes in symptom severity among Black African patients 1 year following UFE for symptomatic uterine fibroids, rated using a validated questionnaire (UFS-QOL). We also aimed to examine changes in health-related quality of life (HRQOL) and potential associations of changes in symptom severity and health-related quality of life with age, parity, uterine volume and fibroid number prior to UFE. Additional interventions after UFE were also recorded. Here we report outcomes 1 year after UFE.

## Methods

This was a prospective observational study over a 1 year period undertaken at the Aga Khan University Hospital Nairobi (AKUH-N) including patients with symptomatic uterine fibroids who had opted for uterine fibroid embolisation (UFE). The patients had presented to the gynaecology clinic with symptomatic fibroid disease and had been offered treatment options including medical approaches, myomectomy (open and laparoscopic), hysterectomy (open and laparoscopic), hysteroscopic resection and UFE after appropriate counselling.

The primary outcome was change in symptom severity using the uterine fibroid symptom and health-related quality of life questionnaire (UFS-QOL) 1 year after the procedure. This questionnaire is available in the Additional file 1. Secondary outcomes examined were changes in HRQOL, and potential associations of changes in symptom severity and health-related quality of life with age, parity, uterine volume and fibroid number prior to UFE. Additional interventions after UFE were recorded with readmission considered as a complication of the procedure. Subsequent myomectomy and hysterectomy were classified as major surgical procedures while hysteroscopy and curettage were classified as minor surgical procedures.

Following approval by the Aga Khan University Research Ethics Committee, participants were enrolled into the study by consecutive selection. Potential participants of Black African descent scheduled for UFE were approached after MR imaging of the pelvis. Exclusions were made on the basis of clinical assessment, relevant clinical investigations and MRI of the uterus and criteria included difficulty in completing English language questionnaires, pathology other than fibroids on MRI, previous UFE, pregnancy, active infection and suspicion of cancer. Patients with a desire to maintain child bearing potential were only recruited after appropriate counseling owing to the uncertain effects of the procedure on ovarian function [10]. Written informed consent was obtained and a validated English language questionnaire instrument, the UFS-QOL [11] was then completed by participants after a brief clinical history. The uterine fibroid symptom and quality of life questionnaire (UFS-QOL) is a disease-specific questionnaire. It assesses symptom severity and HRQOL in patients with uterine fibroids and consists of an 8-item symptom severity scale and 29 HRQOL items covering six domains: Concern, Activities, Energy/Mood, Control, Self-consciousness, and Sexual Function. All items are scored on a 5-point Likert scale, ranging from “not at all” to “a very great deal” for symptom severity items and “none of the time” to “all of the time” for the HRQOL items. Symptom severity and HRQOL subscale scores are summed and transformed into a percentage scale. The symptom severity scale and HRQOL subscale scores are inversely related, with higher symptom severity scores indicating greater symptoms and higher HRQOL subscale scores indicating better HRQOL.

All questionnaires bore a unique participant identification number and names of study participants did not appear on them for purposes of confidentiality. The same questionnaire (UFS-QOL) was administered again after 1 year through email and telephone interviews. Participants were sent reminders after 11 months of the procedure via text messages, telephone calls and email. Follow up for clinical assessment after UFE was undertaken by each patient’s responsible clinician separate from contacts for study data collection.

The uterine volume, volume of the dominant fibroid and number of fibroids prior to UFE were ascertained independently by two radiologists using the pre-UFE pelvic MRI examination. Volumes were determined by measuring the maximum extent of the uterus and dominant leiomyoma in three planes and multiplying the product by 0.5233 (ellipsoid volume formula) [12]. An experienced interventional radiologist (NH) carried out embolisation under conscious sedation using spongostan gelfoam (ETHICON) in the hospital’s cardiac catheterization suite via a transfemoral approach. Pain relief was undertaken using a UFE pain relief protocol previously described [13] and participants were discharged from hospital within 24 h of the procedure and provided with oral pain medication. Participants did not undergo routine follow up MR imaging as previous work in our unit indicated that the extent of shrinkage did not correlate directly with symptomatic improvement [12]. We based our sample size calculation on the outcomes from the FIBROID Registry [14] in which the mean change in symptom severity score was −38.94 (SD ± 24.79), SE 0.52. Given a power of 90% with a 5% significance level, and a null hypothesis value of 70% of the mean change in symptom severity, the sample size was 57 having factored an estimated loss to follow-up of 20%. Data were analysed using STATA version 12 Special Edition and summary statistics tabulated. Statistical significance of differences was tested using t tests, Wilcoxon rank sum tests and Fisher’s exact tests as appropriate taking 5% probability as significant. Age, parity, uterine volume, volume of the dominant fibroid and number of fibroids prior to UFE were categorized into groups for analysis using clinically relevant limits. Potential associations between age, parity, uterine volume, fibroid number prior to UFE and the outcome variables of symptom score change and HRQOL score change were examined using Pearson’s correlation coefficient. Scatter plots with superimposed regression lines were used to illustrate the underlying relationships. Study participants whose data were missing were excluded from the final analysis. However, baseline characteristics for those lost to follow-up were compared to those who completed the study.

## Results

One hundred and thirteen patients undergoing uterine fibroid embolisation (UFE) during the study period were identified as eligible to participate and were approached. Of these, eight declined.

**Fig. 1:**
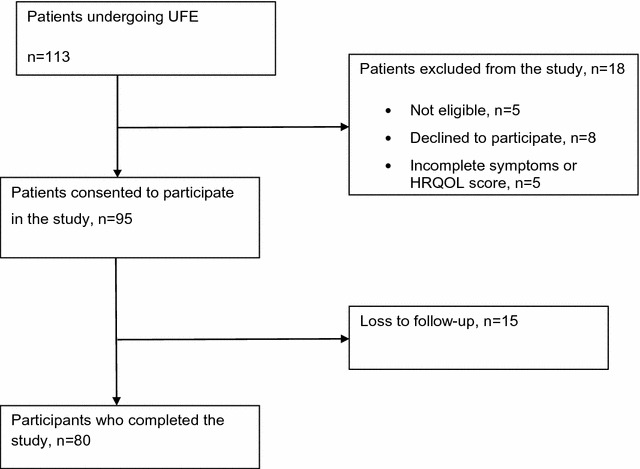
Study profile

Out of the 95 participants who consented to participate in the study, 15 were lost to follow up while 80 (84%) completed follow up and were included in the final analysis (Fig. 1). All participants were able to communicate in the English language. Seventy eight participants completed their follow up questionnaires online while two completed via the telephone. The participants who were lost to follow-up were similar to those who completed the study in terms of age, parity, uterine volume, volume of dominant fibroid, number of fibroids before UFE, symptom severity scores, and HRQOL scores (Additional file 2: Table S1). The characteristics of the participants were skewed and were therefore presented as median and inter quartile range. The median uterine volume (IQR) was 678 ml (486–1121) and the median dominant fibroid volume (IQR) was 124 ml (54–272). Regarding the location of the dominant fibroid, 78% were intramural/transmural, 12% submucosal, and 10% subserosal with majority of participants having at least one submucosal fibroid. Two-thirds of study participants had more than ten fibroids (Additional file 2: Table S1).

### Primary outcome

**Fig. 2:**
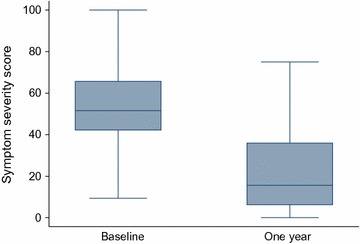
Box and whisker plots of symptom severity scores at baseline and at 1 year

The mean change (improvement) in the symptom severity score was −29.6 (SD ± 27.1) [95% CI −35.6 to −23.6, P < 0.001]. The median symptom score (IQR) at 1 year was 15.6 (6.3–35.9). Sixty eight study participants (85%) recorded an improvement in their symptom severity score. Nine participants (11%) did not record an improvement in either their symptom severity or HRQOL score. Three participants recorded no change in symptom score but had very slight improvement in their health-related quality of life. Seven participants (9%), all of whom were above the age of 40 reported amenorrhoea by 1 year. Figure 2 presents the distribution of symptom severity scores at baseline and at 1 year.

### Secondary outcomes

**Fig. 3:**
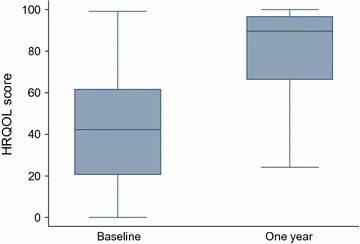
Box and whisker plots of health-related quality of life (HRQOL) scores at baseline and at 1 year

The mean change (improvement) in HRQOL score was 35.7 (SD ± 32.7) [95% CI 28.4 to 42.9, P < 0.001], with significant improvement in all six domains (Additional file 2: Table S2). The median HRQOL score (IQR) at 1 year was 89.7 (66.4–96.6). Figure 3 presents the distribution of HRQOL scores at baseline and at 1 year. There was a strong and significant positive correlation between improvements in symptom severity score and HRQOL (r = 0.76, P < 0.001).

There was a modest but significant correlation between fibroid number before UFE and change in symptom severity score (r_s_ = 0.28, n = 80, P = 0.013). There was no significant correlation between change in symptom severity score and age (r = 0.110, P = 0.332), parity (r = 0.138, P = 0.222) or uterine volume (r = 0.114, P = 0.320). There was a modest statistically significant correlation between parity and change in the health-related quality of life score (r = 0.336, P = 0.002). There was no significant correlation between change in the health-related quality of life score and age (r = 0.109, P = 0.338), uterine volume (r = −0.079, P = 0.492) or fibroid number prior to UFE (r_s_ = 0.16, n = 80, P = 0.166).

During the 1 year follow-up period, 18 (22.5%) study participants required subsequent procedure related care. Two patients on medical therapy eventually required a surgical intervention. The emergency hospitalisation of two participants followed their presentation to the hospital with fever. They were evaluated to exclude infection and post embolization syndrome was diagnosed. They did not require any surgical intervention. Additional file 2: Table S3 illustrates the subsequent care needed for the study participants.

## Discussion

The present study recruited women of Black African descent only so as to reflect the large fibroid burden and associated potential complications in this population. Interestingly, the symptom and HRQOL scores at 1 year in the present study were similar to mean scores of normal subjects reported in the original UFS-QOL validation study. There, normal subjects had a mean symptom severity score of 22.5 (±21.1) and a mean health-related quality of life score of 86.4 (±17.7) [11]. In comparison to the EMMY trial, in which one quarter of the participants were of African descent, participants in our study had twice as large a uterus and dominant fibroid prior to treatment, with similar outcomes in symptom improvement: 84% of participants in the UAE arm of the EMMY trial reported satisfaction after 1 year of the procedure [15]. However, the FIBROID registry in which 48% of study participants were of African descent reported better outcomes than seen here, with a mean change in symptom severity score and HRQOL of 38.9 (95% CI 37.9 to 40.0) and 39.7 (95% CI 38.6 to 40.7) respectively using the UFS-QOL questionnaire at the 12 month follow-up. Of note, a majority of these patients (67.5%) had fewer than five fibroids [14].

We did not observe any association between change in symptom severity score and uterine volume, consistent with an earlier report from our group [12]. However, an interesting observation is that those with a higher number of fibroids prior to UFE have reported a greater improvement in symptoms. This could be due to the likelihood of the presence of more symptom-producing submucosal and intramural fibroids in those with a higher number of fibroids. Other studies have reported conflicting findings in this regard. A prospective study conducted to ascertain if the number, size, or location of fibroids affects therapeutic efficacy or complications of uterine fibroid embolisation concluded that the stated parameters did not impact statistically or clinically on the success rate and the probability of complications [16]. The FIBROID Registry using the UFS-QOL at 3 years evaluated 1916 patients and concluded that smaller leiomyoma size and submucosal location was associated with a greater improvement in symptom severity score [17]. In summary, fibroid number before UFE is more important than uterine volume in predicting symptom outcomes. However, further studies powered to demonstrate this difference need to be carried out before such a conclusive statement can be made. The present study demonstrated a significantly greater improvement in the HRQOL in parous compared to nulliparous women, as also noted in the FIBROID Registry at 1 year follow-up [14]. It is possible that asymptomatic fibroid disease in nulliparous women could have a differential impact in the six domains of the HRQOL namely: Concern, Activities, Energy/Mood, Control, Self-consciousness, and Sexual Function. Overall, advice to women as to whether UFE will improve quality of life needs to reflect individual concerns and perceptions about overall health, preservation of reproductive capacity and fertility as well as the resolution of fibroid symptoms.

Eighteen participants (22.5%) required subsequent care. This is consistent with other reports in the literature: the EMMY trial reported a re-intervention rate (primarily hysterectomy) of 23.5% in the first 2 years [15] while the REST trial reported a surgical re-intervention rate of 32% at 5 years [18]. A retrospective study evaluating long-term clinical and MRI follow up of UFE in patients with a large fibroid burden reported a re-intervention rate of 25% at a mean follow up of 68 months in patients with a large fibroid burden [19]. The majority of our study participants had a submucous fibroid which often requires hysteroscopy to deal with intracavitary myoma debris. This is consistent with our experience that hysteroscopy/dilatation and curettage is the commonest additional procedure needed for these patients. Interestingly and despite this possibility, some studies have demonstrated submucosal location to be a predictor of a greater benefit with regard to symptom severity [14]. In our clinical practice we have debated whether hysteroscopic resection should be routinely offered as an adjunct to UFE for those with submucous disease but as most do not in fact require intervention it would seem reasonable to defer hysteroscopy unless there are symptoms suggestive of retained degenerating tissue.

The finding of increased risk of amenorrhoea with age following UFE is consistent with other reports and may be due to a higher susceptibility of ovarian tissue to ischaemic insult as a feature of reproductive ageing [10, 17, 20]. Therefore, women who wish to maintain fertility potential should be counselled fully regarding the risk of amenorrhoea in the context of their individual circumstances.

The introduction of a non-surgical modality for treating fibroids is very attractive in the African setting where the condition is very common and a cause of substantial interference to normal function and quality of life through intractable heavy menstrual bleeding leading to severe anemia or extreme pressure symptoms. Certain components of safe surgery especially access to blood transfusion are frequently challenging in the regional context. Cost and the infrastructure necessary to undertake UFE in a systematic way naturally require careful consideration. Regarding the latter, there are synergies with other emerging clinical service developments such as cardiac catheterization laboratories and the availability of this non-surgical modality of fibroid treatment should increase as the capacity for interventional cardiology increases. For the procedure itself, the use of the spongostan gelfoam (ETHICON) for embolisation rather than the more costly embolic beads and deferring routine contrast-enhanced MRI pelvis to predict future fibroid regrowth has enabled the cost containment while maintaining efficacy and safety. Overall, the cost of UFE can be considered comparable to that of surgery [21] but further work is needed in the region to extend interventional radiology training, access to appropriate facilities and careful co-ordination of referral and care across specialists in radiology and gynaecology in order to render this valuable modality accessible to those in need.

## Conclusions

We conclude that UFE is associated with a clinically useful and statistically significant symptom relief in Black African patients. Women considering the procedure can be advised that symptom improvement following UFE is not compromised by a large fibroid burden and the rate of subsequent intervention is within an acceptable range. Furthermore, the number of fibroids is potentially more significant than the volume of the uterus in predicting likely outcomes.

## Limitations

First, 16% of study participants did not complete the post-procedure questionnaire at 12 months. Thus, the results would have been biased towards favourable outcomes among UFE patients if these study participants were more likely to experience unfavourable outcomes. The similarity of baseline characteristics of those completing and not completing follow up is however reassuring. Secondly, pharmacological interventions were not considered prior to administration of the questionnaires either before or after UFE. These pharmacological interventions had the potential of altering the symptom and HRQOL scores.

## Future research

The influence of pre procedure fibroid number on symptom improvement following UFE.

Risk factors for amenorrhoea following UFE.

## Additional files

**Additional file 1.** Uterine fibroid symptom and health-related quality of life questionnaire.

**Additional file 2: Table S1.** Characteristics of study participants who completed the study and those lost to follow up. **Table S2.** Changes in the health-related quality of life domains. **Table S3.** Subsequent Care.
