# Supplementary material for: One year symptom severity and health-related quality of life changes among Black African patients undergoing uterine fibroid embolisation
Source: BMC Res Notes. 2017 Jul 4;10:240. doi: 10.1186/s13104-017-2558-0 (PMC5496397; doi:10.1186/s13104-017-2558-0)
Supplement: Supplementary file 1 — Additional file 1. Uterine fibroid symptom and health-related quality of life questionnaire. [file 13104_2017_2558_MOESM1_ESM.docx]

Additional file 1

**UTERINE FIBROID SYMPTOM AND HEALTH-RELATED QUALITY OF LIFE QUESTIONNAIRE**

**(UK English version of the UFS-QOL)**

**Listed below are symptoms experienced by women who have uterine fibroids. Please consider each symptom as it relates to your uterine fibroids or menstrual cycle. Each question asks how much distress you have experienced from each symptom during the last 3 months.**

| X |
| --- |

There are no right or wrong answers. Please be sure to **answer every question** by crossing

the most appropriate box for you. If a question does not apply to you, please mark "not at all" as a response.

During the **last 3 months**, how distressed were you by…

Not at all

A little bit

Some- what

A great deal

A very great deal

|  |
| --- |

|  |
| --- |

|  |
| --- |

|  |
| --- |

|  |
| --- |

1. Heavy bleeding during your menstrual period?

1 2 3 4 5

|  |
| --- |

|  |
| --- |

|  |
| --- |

|  |
| --- |

|  |
| --- |

2. Passing blood clots during your menstrual

period?

1 2 3 4 5

|  |
| --- |

|  |
| --- |

|  |
| --- |

|  |
| --- |

|  |
| --- |

3. Variations in the length of your menstrual

periods?

1 2 3 4 5

|  |
| --- |

|  |
| --- |

|  |
| --- |

|  |
| --- |

|  |
| --- |

4. Variations in the number of days between each

menstrual period?

1 2 3 4 5

|  |
| --- |

|  |
| --- |

|  |
| --- |

|  |
| --- |

|  |
| --- |

5. Feeling tightness or pressure in your pelvic area

(lower part of the belly)?

1 2 3 4 5

|  |
| --- |

|  |
| --- |

|  |
| --- |

|  |
| --- |

|  |
| --- |

6. Frequent urination during the daytime?

1 2 3 4 5

|  |
| --- |

|  |
| --- |

|  |
| --- |

|  |
| --- |

|  |
| --- |

7. Frequent night-time urination?

1. 2 3 4 5

|  |
| --- |

|  |
| --- |

|  |
| --- |

|  |
| --- |

|  |
| --- |

8. Feeling tired?

1 2 3 4 5

 Copyright 2001 CIRREF. All rights reserved 1

f:\institut\cultadap\project\hc1617\question\uk english\final\ufsukq.doc-21/12/2001

The following questions ask about your feelings and experiences regarding the impact of uterine fibroid symptoms on your life. Please consider each question as it relates to your experiences with uterine fibroids during the **last 3 months.**

There are no right or wrong answers. Please be sure to answer every question by crossing **(**X**)** the most appropriate box for you. If the question does not apply to you, please tick **"**none of the time**"** as your option.

**During the last 3 months**, how often have your symptoms related to uterine fibroids...

None of the time

A little of the time

Some of the time

Most of the time

All of the time

|  |
| --- |

|  |
| --- |

|  |
| --- |

|  |
| --- |

|  |
| --- |

9. Made you worry because you did not know when your period would start or how long it

would last? 1 2 3 4 5

|  |
| --- |

|  |
| --- |

|  |
| --- |

|  |
| --- |

|  |
| --- |

10. Made you anxious about travelling?

1. 2 3 4 5

|  |
| --- |

|  |
| --- |

|  |
| --- |

|  |
| --- |

|  |
| --- |

11. Interfered with your physical activities?

1. 2 3 4 5

|  |
| --- |

|  |
| --- |

|  |
| --- |

|  |
| --- |

|  |
| --- |

12. Caused you to feel tired or worn out?

1. 2 3 4 5

|  |
| --- |

|  |
| --- |

|  |
| --- |

|  |
| --- |

|  |
| --- |

13. Made you spend less time on exercise or

other physical activities?

1 2 3 4 5

|  |
| --- |

|  |
| --- |

|  |
| --- |

|  |
| --- |

|  |
| --- |

14. Made you feel as if you are not in control of

your life?

1 2 3 4 5

|  |
| --- |

|  |
| --- |

|  |
| --- |

|  |
| --- |

|  |
| --- |

15. Made you concerned about soiling your

underwear?

1 2 3 4 5

|  |
| --- |

|  |
| --- |

|  |
| --- |

|  |
| --- |

|  |
| --- |

16. Made you feel less productive?

1 2 3 4 5

|  |
| --- |

|  |
| --- |

|  |
| --- |

|  |
| --- |

|  |
| --- |

17. Caused you to feel drowsy or sleepy during

the day?

1 2 3 4 5

|  |
| --- |

|  |
| --- |

|  |
| --- |

|  |
| --- |

|  |
| --- |

18. Made you feel self-conscious of weight gain?

1. 2 3 4 5

**During the last 3months**, how often have your None of A little Some Most of All of

symptoms related to uterine fibroids……. the time of the of the of the the

time time time time

|  |
| --- |

|  |
| --- |

|  |
| --- |

|  |
| --- |

|  |
| --- |

19. Made you feel that it was difficult to carry

out your usual activities?

1 2 3 4 5

|  |
| --- |

|  |
| --- |

|  |
| --- |

|  |
| --- |

|  |
| --- |

20. Interfered with your social activities (e.g., going out to the cinema, restaurants, parties,

etc)? 1 2 3 4 5

|  |
| --- |

|  |
| --- |

|  |
| --- |

|  |
| --- |

|  |
| --- |

21. Made you feel conscious about the size and

appearance of your stomach?

1 2 3 4 5

|  |
| --- |

|  |
| --- |

|  |
| --- |

|  |
| --- |

|  |
| --- |

22. Made you concerned about soiling bed linen? 1 2 3 4 5

1 2 3 4 5

|  |
| --- |

|  |
| --- |

|  |
| --- |

|  |
| --- |

|  |
| --- |

23. Made you feel sad, discouraged, or hopeless?

1 2 3 4 5

1 2 3 4 5

|  |
| --- |

|  |
| --- |

|  |
| --- |

|  |
| --- |

|  |
| --- |

24. Made you feel down-hearted and blue?

1 2 3 4 5

|  |
| --- |

|  |
| --- |

|  |
| --- |

|  |
| --- |

|  |
| --- |

25. Made you feel exhausted?

1. 2 3 4 5

|  |
| --- |

|  |
| --- |

|  |
| --- |

|  |
| --- |

|  |
| --- |

26. Caused you to be concerned or worried about

your health?

1 2 3 4 5

|  |
| --- |

|  |
| --- |

|  |
| --- |

|  |
| --- |

|  |
| --- |

27. Caused you to plan activities more carefully?

1. 2 3 4 5

|  |
| --- |

|  |
| --- |

|  |
| --- |

|  |
| --- |

|  |
| --- |

28. Made you feel inconvenienced by always

having to carry extra pads, tampons, and

clothing in case of accidents? 1 2 3 4 5

|  |
| --- |

|  |
| --- |

|  |
| --- |

|  |
| --- |

|  |
| --- |

29. Caused you embarrassment?

1. 2 3 4 5

|  |
| --- |

|  |
| --- |

|  |
| --- |

|  |
| --- |

|  |
| --- |

30. Made you feel uncertain about your future?

1 2 3 4 5

1 2 3 4 5

**During the last 3months**, how often have your None of A little Some Most of All of

symptoms related to uterine fibroids……. the time of the of the of the the

time time time time

|  |
| --- |

|  |
| --- |

|  |
| --- |

|  |
| --- |

|  |
| --- |

31. Made you feel irritable?

1 2 3 4 5

|  |
| --- |

|  |
| --- |

|  |
| --- |

|  |
| --- |

|  |
| --- |

32. Made you concerned about soiling your outer

clothes?

1 2 3 4 5

|  |
| --- |

|  |
| --- |

|  |
| --- |

|  |
| --- |

|  |
| --- |

33. Affected the size of clothing you wear during

your periods?

1 2 3 4 5

|  |
| --- |

|  |
| --- |

|  |
| --- |

|  |
| --- |

|  |
| --- |

34. Made you feel that you are not in control of

your health?

1 2 3 4 5

|  |
| --- |

|  |
| --- |

|  |
| --- |

|  |
| --- |

|  |
| --- |

35. Made you feel weak as if energy was drained

from your body?

1 2 3 4 5

|  |
| --- |

|  |
| --- |

|  |
| --- |

|  |
| --- |

|  |
| --- |

36. Decreased your sex drive?

1. 2 3 4 5

|  |
| --- |

|  |
| --- |

|  |
| --- |

|  |
| --- |

|  |
| --- |

37. Caused you to avoid sexual relations?

1 2 3 4 5

1 2 3 4 5
