# Supplementary material for: One year symptom severity and health-related quality of life changes among Black African patients undergoing uterine fibroid embolisation
Source: BMC Res Notes. 2017 Jul 4;10:240. doi: 10.1186/s13104-017-2558-0 (PMC5496397; doi:10.1186/s13104-017-2558-0)
Supplement: Supplementary file 2 — Additional file 2: Table S1. Characteristics of study participants who completed the study and those lost to follow up. Table S2. Changes in the health-related quality of life domains. Table S3. Subsequent Care. [file 13104_2017_2558_MOESM2_ESM.docx]

**Additional file 2**

***Table S1: Characteristics of study participants who completed the study and those lost to follow up***

| **Variable** | **LOSS TO**  **FOLLOW-UP (n=15)** | | **STUDY PARTICIPANTS**  **(n=80)** | | **Test for differences** |
| --- | --- | --- | --- | --- | --- |
|  | **Median** | **IQR** | **Median** | **IQR** | **P** |
| **Age at procedure (years)** | 44.0 | 39.0-48.0 | 43.0 | 39.0-47.0 | 0.642 |
| **Parity** | 2.0 | 0.0-4.0 | 1.0 | 0.0-2.0 | 0.273 |
| **^++^Volume of Dominant Fibroid(mls)** | 192.0 | 65.0-372.0 | 124.0 | 54.0-272.0 | 0.545 |
| **^+^Uterine Volume at procedure(mls)** | 664.0 | 467.0-986.0 | 677.5 | 486.0-1121.0 | 0.762 |
| **Baseline Symptom severity score** | 59.4 | 31.3-65.6 | 51.6 | 42.2-65.6 | 0.748 |
| **Baseline HRQoL score** | 48.3 | 26.7-65.5 | 42.2 | 20.7-61.6 | 0.414 |
| **Number of Fibroids before UFE** | **n** | **%** | **n** | **%** |  |
| **1-10** | 11 | 73.3% | 27 | 34% |  |
| **11-20** | 2 | 13.3% | 32 | 40% | 0.072^f^ |
| **21-30** | 2 | 13.3% | 15 | 19% |  |
| **31-40** | 0 |  | 5 | 6% |  |
| **>40** | 0 |  | 1 | 1% |  |

^+^ - Sample size=78 among those who completed the study

^++^ - Sample size=73 among those who completed the study

^f^ - Fisher’s exact test

***Table S2: Changes in the health-related quality of life domains***

| **Domain** | **Mean (SD)** | **95% CI** | **P** |
| --- | --- | --- | --- |
| Concern | 38.5(41.3) | 29.3 - 47.7 | <0.0001 |
| Activities | 39.1(36.4) | 31.0 - 47.1 | <0.0001 |
| Energy/Mood | 34.4(33.9) | 26.8 - 41.9 | <0.0001 |
| Control | 37.3(35.3) | 29.4 - 45.1 | <0.0001 |
| Self-conscious | 32.5(36.4) | 24.4 - 40.6 | <0.0001 |
| Sexual Function | 26.4(40.4) | 17.4 - 35.4 | <0.0001 |

***Table S3: Subsequent Care***

| **MODALITY** |  | **N=80** |  |
| --- | --- | --- | --- |
|  |  | n | % |
| Major surgical procedure | Hysterectomy | 1 | 1.25 |
|  | Myomectomy | 4 | 5.0 |
| Minor surgical procedure | Hysteroscopy | 9 | 11.25 |
|  | Dilatation and curettage | 1 | 1.25 |
| Emergency hospitalization |  | 2 | 2.5 |
| Medical therapy | Progesterone (LNG-IUS) | 1 | 1.25 |
|  | Tranexamic acid | 2 | 2.5 |
